# High-Flow Nasal Oxygen Therapy in Preventing Post-Extubation Hypoxaemia and Postoperative Pulmonary Complications: A Systematic Review and Meta-Analysis †

**DOI:** 10.3390/diagnostics15192449

**Published:** 2025-09-25

**Authors:** Jamie Wen Yen Tan, Azarinah Izaham, Raha Abd Rahman, Rufinah Teo, Syarifah Noor Nazihah Sayed Masri, Azrina Md Ralib, Kok-Yong Chin

**Affiliations:** 1 Department of Anaesthesiology and Intensive Care, Faculty of Medicine, Universiti Kebangsaan Malaysia, Jalan Yaacob Latif, Bandar Tun Razak, Cheras, Kuala Lumpur 56000, Malaysia; tanwenyen@gmail.com (J.W.Y.T.); raharahman1504@gmail.com (R.A.R.); rufinah@hctm.ukm.edu.my (R.T.); 2 Department of Anaesthesiology and Intensive Care, Hospital Canselor Tuanku Muhriz, Universiti Kebangsaan Malaysia, Jalan Yaacob Latif, Bandar Tun Razak, Cheras, Kuala Lumpur 56000, Malaysia; syarifahnoornazihah@gmail.com; 3 Department of Anaesthesiology and Intensive Care, Kulliyyah of Medicine International Islamic University of Malaysia, Bandar Indera Mahkota, Kuantan 25200, Malaysia; azrinar@iium.edu.my; 4 Department of Pharmacology, Faculty of Medicine, Universiti Kebangsaan Malaysia, Jalan Yaacob Latif, Bandar Tun Razak, Cheras, Kuala Lumpur 56000, Malaysia; chinkokyong@ppukm.ukm.edu.my

**Keywords:** high-flow nasal oxygen, postoperative, hypoxaemia, extubation, pulmonary complications

## Abstract

**Background**: Post-extubation hypoxaemia and postoperative pulmonary complications (PPCs) are common in surgical patients and contribute significantly to morbidity and prolonged recovery. High-flow nasal oxygen therapy (HFNOT) has been proposed as an alternative to conventional oxygen therapy (COT) in improving oxygenation and reducing PPCs postoperatively. **Objectives**: To evaluate the effectiveness of HFNOT compared to COT in reducing post-extubation hypoxaemia and PPCs in adult surgical patients, and to assess its impact on other clinical outcomes including ICU and hospital length of stay, mortality, and the need for escalation of respiratory support. **Methods**: A systematic review and meta-analysis of randomized controlled trials was conducted following PRISMA guidelines. Studies were identified from five databases including PubMed, Scopus, EBSCOHost, ProQuest, Ovid MEDLINE and Web of Science. Adult postoperative patients who received HFNOT after extubation were compared to those receiving COT. Primary outcomes included PaO_2_/FiO_2_ (PF) ratio and incidence of PPCs. Secondary outcomes were hospital and ICU length of stay, mortality, and need for escalation of therapy. **Results**: Seventeen trials comprising 1830 patients were included. HFNOT significantly improved PF ratio post-extubation and reduced the incidence of hypoxaemia and PPCs compared to COT. For secondary outcomes, HFNOT was associated with a reduced hospital length of stay and lower postoperative mortality, while no significant difference was found for ICU stay. Escalation of respiratory support was more frequent in the COT group. Subgroup analyses indicated greater improvements in oxygenation with HFNOT of shorter duration (<24 h) and in non-cardiothoracic patients. **Conclusions**: HFNOT is associated with improved postoperative oxygenation and a reduction in respiratory complications following extubation in surgical patients. The most pronounced benefits were observed in non-cardiothoracic populations and with short-duration applications. While the beneficial effects of HFNOT appear consistent across the included randomized controlled trials, further large-scale studies with standardized intervention durations, surgical populations, and clearly defined criteria for escalation of therapy are needed to strengthen and confirm these findings.

## 1. Introduction

The aetiology of postoperative pulmonary complications (PPCs) is complex and multifactorial, involving both the effects of general anaesthesia and the physiological impact of surgery [1]. General anaesthesia induces mechanical and functional changes in the respiratory system, including a reduction in functional residual capacity and the formation of atelectasis in dependent lung regions [2]. Certain surgical procedures confer a higher risk of PPCs, with abdominal and vascular surgeries, particularly abdominal aortic aneurysm repair, being consistently associated with elevated risk. This is followed by thoracic, upper abdominal, neck and neurosurgical procedures [3].

Post-extubation hypoxaemia, one of the most frequent postoperative complications, arises from a broad range of pathophysiological mechanisms, including pneumonia, atelectasis, bronchospasm, pleural effusion, pulmonary embolism and ventilatory depression [4,5]. It is strongly associated with adverse patient outcomes, including prolonged hospital stay, increased healthcare costs and up to an 18-fold increase in 30-day mortality [6,7,8,9]. Conventional oxygen therapy (COT) via low-flow nasal cannula, simple face mask or Venturi mask is commonly used to mitigate hypoxaemia in the immediate postoperative period. However, these modalities may not provide sufficient respiratory support in all cases. Escalation to non-invasive ventilation (NIV), such as continuous positive airway pressure (CPAP) or bilevel positive airway pressure (BiPAP), or even invasive mechanical ventilation, is often required. These approaches are resource-intensive and may not be well tolerated by conscious patients. In addition to that, a systematic review published in 2014 by Ireland et al. found that although CPAP initiated during the postoperative period might reduce atelectasis, pneumonia and reintubation, its effects on mortality, hypoxia or invasive ventilation remain uncertain [10]. Moreover, NIV frequently causes patient discomfort and often necessitates ICU monitoring, thereby increasing the risk of ICU-related complications and prolonging hospitalization [11]. High-flow nasal oxygen therapy (HFNOT) has emerged as a promising alternative, particularly within the ICU and during the COVID-19 pandemic. Capable of delivering heated, humidified oxygen at flow rates up to 60 L/min with a high fraction of inspired oxygen (FiO_2_), HFNOT is generally well tolerated in conscious patients and easy to administer. Its physiological benefits include improved oxygenation, enhanced washout of anatomical dead space, better mucociliary clearance and airway humidification [12,13]. In addition, the generation of low-level positive end-expiratory pressure (PEEP) in the upper airway contributes to alveolar recruitment and reduced work of breathing [14]. While HFNOT is well established for managing acute hypoxaemic respiratory failure in the ICU [15], its role in preventing post-extubation hypoxaemia in surgical patients remains less well defined. Evidence of its effectiveness in reducing the need for escalation to respiratory support or reintubation in the postoperative setting is still emerging. Given its physiological advantages and clinical potential, it is plausible that HFNOT could help mitigate PPCs in this population. Therefore, a systematic review and meta-analysis is warranted to evaluate the current evidence on the use of HFNOT in the postoperative period.

## 2. Materials and Methods

### 2.1. Protocol and Registration

The systematic review and meta-analysis was conducted in accordance with the recommendation of the Preferred Reporting Items for Systematic Review and Meta-analyses (PRISMA) statement (please see the PRISMA checklist in Supplementary Materials), and the protocol was registered on PROSPERO (ID: CRD 42023403246).

### 2.2. Data Sources and Searches

PubMed, Scopus, ProQuest, Ovid MEDLINE and Web of Science databases were comprehensively searched from inception to 12 July 2025 using the following keywords: (“High Flow Nasal Cannula” OR “high flow nasal oxygen” OR “high-flow nasal cannula” OR “HFNO” OR “HFNC” OR “heated humidified high-flow”) AND (“Postoperative Complications” [Mesh] OR “postoperative pulmonary complications” OR “post-extubation” OR “post extubation” OR “weaning from mechanical ventilation”) AND (“Hypoxia” [Mesh] OR hypoxemia OR hypoxaemia OR “oxygen desaturation” OR “respiratory failure” OR “reintubation” OR “pulmonary complications” OR pneumonia OR atelectasis). Additional studies were identified by manually searching the reference lists from relevant articles and reviews. Covidence and MS Excel were used for managing the searched literature.

### 2.3. Inclusion and Exclusion Criteria

The eligibility criteria for included trials are listed below by the population, intervention, comparison, outcomes and study design (PICOS) strategy: (a) Population: Post-operative adult surgical patients of any gender aged 18 years and above. This study did not limit inclusion to any specific type of disease or surgery. (b) Intervention: The application of high-flow nasal oxygen therapy after extubation. (c) Comparison: The use of conventional oxygen therapy (low-flow nasal prongs, simple face mask, venturi mask, and high-flow face mask) in control population. (d) Outcomes: Primary outcomes include PaO_2_/FiO_2_ ratios within the study period and the incidence of postoperative pulmonary complications. Secondary outcomes include ICU length of stay, hospital length of stay, mortality rates and rate of escalation of therapy. (e) Study design: Randomized Controlled Trials.

Articles that were excluded are studies published as reviews, letters, editorials, case reports, protocols, crossover trials, or retrospective, observational, cohort or case–control studies. Pre-clinical studies and non-English language papers were also excluded.

### 2.4. Definition

HFNOT was defined as high-flow nasal cannula delivering up to 100% heated and humidified oxygen at a flow rate of 25 up to 60 L/min. COT was defined as oxygen delivered via low-flow nasal cannula, simple face mask, Venturi mask or rebreathing and non-rebreathing high-flow face mask. The definition for reintubation was intubation of the trachea within the individual study period after post-operative extubation. Postoperative pulmonary complications were defined according to the European Perioperative Clinical Outcome (EPCO) criteria, which comprise a composite of clinical conditions including respiratory infection, respiratory failure, pleural effusion, atelectasis, pneumothorax, bronchospasm and aspiration pneumonitis [16].

### 2.5. Study Selection

Three review authors (JWYT, AI, AMR) independently assessed titles and abstracts to determine the suitability of studies based on inclusion and exclusion criteria. Full texts of potentially relevant studies were then retrieved and reviewed. A third reviewer (RAR) adjudicated disagreements on study selections.

### 2.6. Data Extraction

Three review authors (JWYT, AI, AMR) independently extracted data from the included studies using a data template. The data extracted include the first author’s name, year of publication, study population, country of origin, study design, number of patients, patients’ baseline characteristics, type of oxygen therapy administered and outcome parameters.

### 2.7. Risk of Bias Assessment and Quality of Evidence

Risk of bias of the included randomised controlled trials (RCTs) was assessed using the 2019 Cochrane Collaboration methodology (ROB2), which evaluates the following domains: randomization process, deviations from intended interventions, missing outcome data, measurement of outcome, selection of reported result and overall risk of bias judgment. The studies were reported as low-risk, some concerns or high-risk.

### 2.8. Statistical Analysis

Statistical analysis was conducted using Review Manager (RevMan) Version 5.3. Continuous outcomes were reported as mean differences (MDs), and dichotomous outcomes were assessed using odds ratios (ORs), both with corresponding 95% confidence intervals (CIs). When continuous data were presented as medians with interquartile ranges (IQRs), conversion to means and standard deviations were performed using the formula recommended by the Cochrane Collaboration via the online Meta-Analysis Accelerator tool [17,18]. Heterogeneity among studies was evaluated using the Chi-squared (χ^2^) test and I^2^ statistic, with thresholds of 25%, 50% and 75% representing low, moderate and high heterogeneity, respectively. All analyses were conducted according to the intention-to-treat principle. A *p*-value < 0.05 was considered statistically significant. Results were visually summarized using Forest plots.

Subgroup analyses were performed to explore heterogeneity and identify patient populations most likely to benefit from HFNOT. Subgroups were stratified based on the duration of HFNOT vs. COT application and type of surgery (cardiothoracic vs. non-cardiothoracic procedures).

## 3. Results

### 3.1. Trial Selection

**Figure 1:**
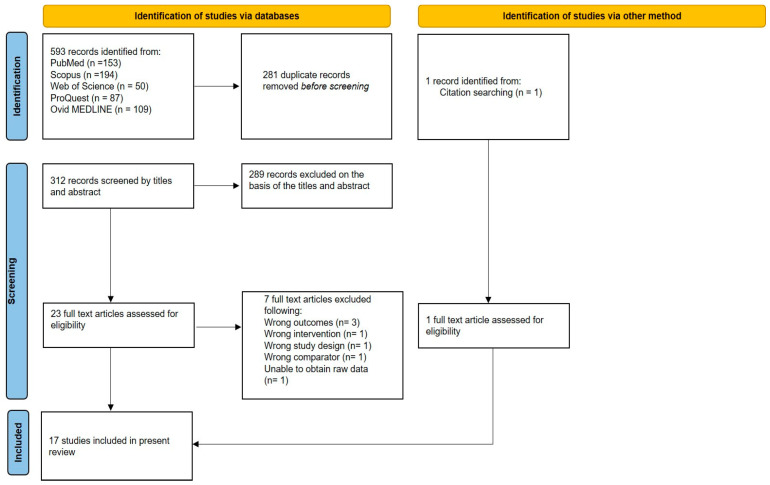
PRISMA flow diagram of literature search.

A total of 593 potentially relevant studies were identified through the systematic literature search. After the removal of 281 duplicates, 312 studies were screened by title and abstract. Of these, 289 were excluded based on predefined eligibility criteria. The remaining 23 full-text articles were assessed for inclusion, with one additional study identified through citation searching. Following full-text review, 7 studies were excluded, resulting in the inclusion of 17 trials in the final analysis. The study selection process is detailed in Figure 1.

### 3.2. Trial Characteristics

The baseline characteristics of the included trials and details of their respective interventions are summarized in Table 1 and Table 2. The studies were published between 2013 and 2024, with sample sizes ranging from 34 to 340 patients. Six trials focused on patients undergoing cardiac surgery, three each on laparoscopic bariatric surgery, thoracic and abdominal surgeries, and one trial each on laparoscopic gynaecological and orthopaedic surgeries.

The target SpO_2_ was defined as the minimum oxygen saturation threshold at which escalation of respiratory support would be initiated. The duration of the study interventions ranged from 1 h to 5 days postoperatively. All included trials compared HFNOT with COT, delivered via nasal prongs, face mask, Venturi mask or high-flow face mask.

In all studies, HFNOT was administered prophylactically, either following a predefined therapeutic protocol or at the discretion of the attending intensivist or primary physician.

### 3.3. Risk of Bias Assessment

**Figure 2:**
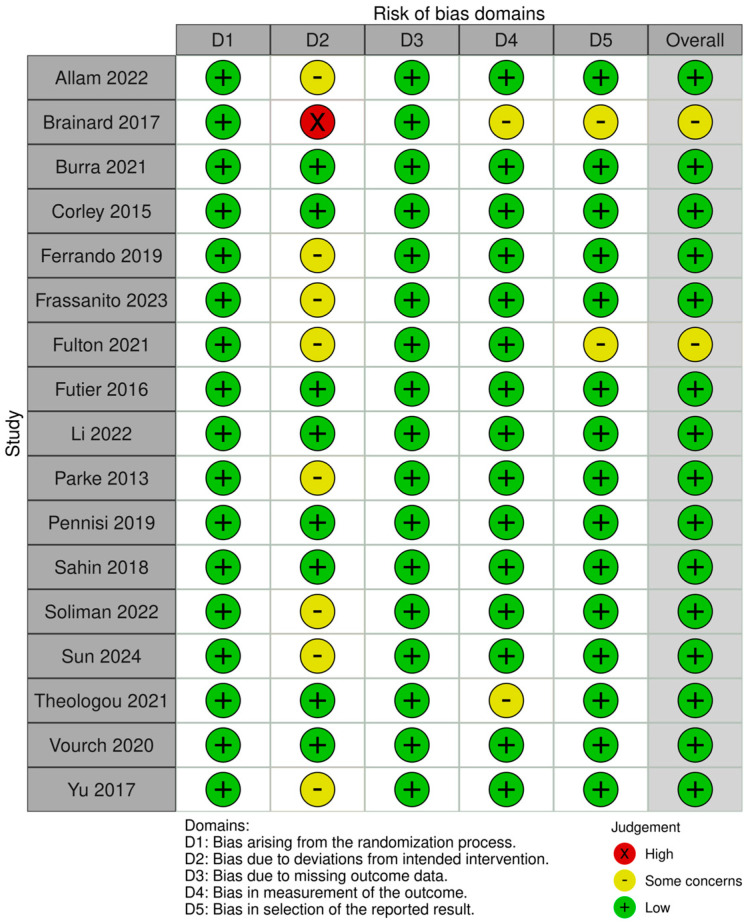
Summary of Risk of Bias [19,20,21,22,23,24,25,26,27,28,29,30,31,32,33,34,35,36].

The methodological quality of the included RCTs was assessed using the Cochrane Risk of Bias 2.0 tool, focusing on five domains, and is summarized in Figure 2. Of the 17 studies, the majority were judged to have low risk of bias in the randomization process (D1), data completeness (D3), outcome measurement (D4) and risk of selective reporting (D5). Deviations from intended interventions (D2) were frequent sources of “some concerns”, primarily due to lack of blinding from the patient and the intensivist/primary physician in view of the visibility of apparatus.

### 3.4. Outcomes

#### 3.4.1. Primary Outcomes

Seven trials reported the PF ratio at the end of the intervention period, involving a total of 282 patients in both the HFNOT and COT groups. The meta-analysis showed that the PF ratio was significantly higher in patients who received HFNOT compared to those who received COT (mean difference [MD] 21.09, 95% confidence interval [CI] 12.72 to 29.47; *p* < 0.00001).

**Figure 3:**
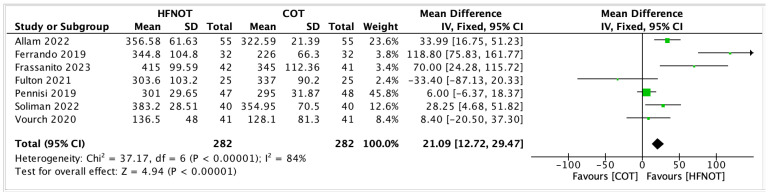
Forest plot showing the postoperative PF ratio of patients receiving HFNOT vs. COT [19,23,24,25,29,31,34].

As illustrated in Figure 3, the majority of individual studies demonstrated a positive effect of HFNOT on oxygenation, with several showing statistically significant improvements [19,23,24]. One study by Fulton et al. reported a mean difference in favour of COT, although the confidence interval crossed the line of no effect, indicating statistical non-significance [25].

Despite the overall benefit, there was substantial heterogeneity across the studies (I^2^ = 84%, χ^2^ = 37.17, *p* < 0.00001), possibly attributable to variations in surgical populations, timing of PF ratio measurement, and HFNOT protocols used.

**Figure 4:**
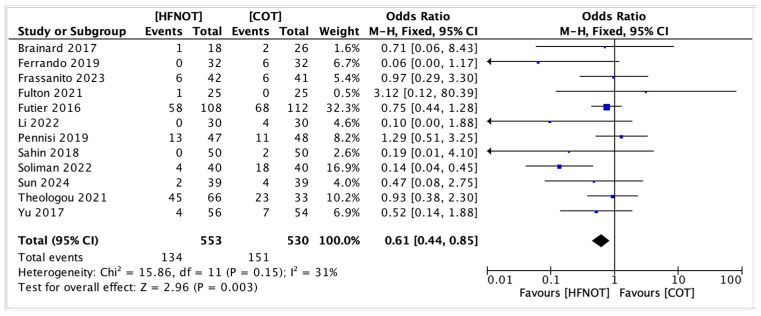
Forest plot showing the incidence of PPCs in patients receiving HFNOT vs. COT [20,23,24,25,26,27,29,30,31,32,33,35].

Twelve studies were included in the meta-analysis for incidence of PPCs, involving 553 patients in the high-flow nasal oxygen therapy (HFNOT) group and 530 in the conventional oxygen therapy (COT) group. Pooled analysis as illustrated in Figure 4 showed that HFNOT significantly reduced the odds of the adverse outcome compared to COT, with an overall odds ratio (OR) of 0.61 (95% CI: 0.44–0.85, *p* = 0.003).

Significant benefits were observed in several studies, including Soliman et al. (OR: 0.14; 95% CI: 0.04–0.45), Sun et al. (OR: 0.47; 95% CI: 0.25–0.89), and Yu et al. (OR: 0.52; 95% CI: 0.14–1.88) [31,32,35]. Other studies showed non-significant effects, with confidence intervals crossing unity.

Heterogeneity was low to moderate (χ^2^ = 15.86, *df* = 11, *p* = 0.15; *I*^2^ = 31%), supporting the use of a fixed-effects model. The overall effect was statistically significant (*Z* = 2.96, *p* = 0.003), indicating a consistent advantage of HFNOT over COT.

#### 3.4.2. Secondary Outcomes

1. ICU Length of Stay

**Figure 5:**
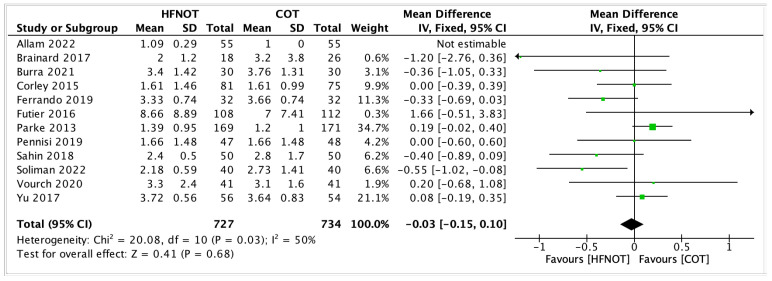
ICU Length of Stay [19,20,21,22,23,26,28,29,30,31,34,35].

As shown in Figure 5, twelve trials reported on the length of ICU stay in patients receiving HFNOT versus COT. The meta-analysis revealed no significant difference between the two groups (MD = −0.03, 95% CI −0.15–0.10, *p* = 0.68). Moderate heterogeneity was observed among the studies (I^2^ = 50%, χ^2^ = 20.08, *p* = 0.03).

Most trials reported small differences between HFNOT and COT, with confidence intervals crossing the line of no effect. Only two studies by Futier et al. and Parke et al. showed statistically significant results in favour of HFNOT, while others demonstrated neutral effects [26,28]. These findings suggest that prophylactic HFNOT does not significantly reduce ICU length of stay compared to conventional oxygen therapy in the postoperative population studied.

2 Hospital Length of Stay

**Figure 6:**
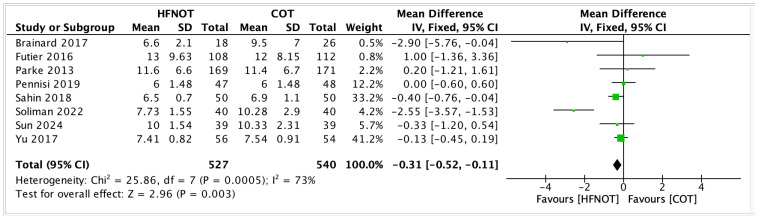
Hospital Length of Stay [20,26,28,29,30,31,32,35].

Eight trials reported on hospital length of stay in patients receiving HFNOT (*n* = 527) versus COT (*n* = 540). The meta-analysis demonstrated a statistically significant reduction in hospital stay for patients treated with HFNOT (MD—0.31 days, 95% CI −0.52 to −0.11; *p* = 0.003) (Figure 6).

Despite the overall benefit, substantial heterogeneity was observed among studies (I^2^ = 73%, χ^2^ = 25.86; *p* = 0.0005).

**Figure 7:**
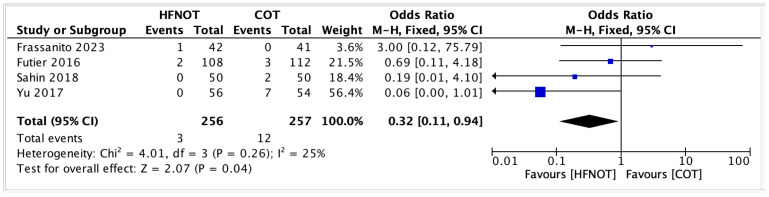
Postoperative mortality rates [24,26,30,35].

3 Mortality rate
  Four trials reported on postoperative mortality in patients receiving HFNOT (*n* = 256) versus COT (*n* = 257). The pooled analysis showed a significantly lower odds of mortality in the HFNOT group (OR 0.32, 95% CI 0.11 to 0.94; *p* = 0.04) (Figure 7).
  A total of 3 deaths occurred in the HFNOT group compared to 12 deaths in the COT group. Heterogeneity was low (I^2^ = 25%, χ^2^ = 4.01; *p* = 0.26), indicating consistency across studies.

4 Escalation of respiratory support
  Escalation of respiratory support was mainly performed under the discretion of individual intensivists/primary physicians unless a therapy algorithm was in place. Table 3 summarizes escalation events from initial oxygen therapy to more advanced support modalities—including escalation to high-flow nasal oxygen therapy (HFNOT), non-invasive ventilation (NIV), and reintubation—across included studies. Total escalation refers to the combined number of patients requiring any of these interventions. Overall, escalation events were more frequent in the COT group compared to HFNOT, particularly for transitions to NIV or reintubation (Table 3).

### 3.5. Subgroup Analyses

Subgroup analyses were performed to explore potential sources of heterogeneity in the PF ratio outcome, based on duration of HFNOT and type of surgery (Table 4). In studies where HFNOT was administered for less than 24 h, the PF ratio was significantly higher compared to COT (MD 42.45, 95% CI 27.92–56.97; *p* < 0.00001), although heterogeneity was substantial (I^2^ = 86%). In contrast, studies with HFNOT duration exceeding 24 h also demonstrated a significant but smaller improvement in PF ratio (MD 10.51, 95% CI 0.26–20.75; *p* = 0.04) with low heterogeneity (I^2^ = 26%).

When stratified by surgical type, patients undergoing non-cardiothoracic surgeries showed a significantly greater improvement in PF ratio with HFNOT compared to COT (MD 38.50, 95% CI 26.13–50.88; *p* < 0.00001), with high heterogeneity (I^2^ = 83%). However, in the cardiothoracic surgery subgroup, no significant difference was observed between HFNOT and COT (MD 6.37, 95% CI −5.00 to 17.75; *p* = 0.27) and no heterogeneity was detected (I^2^ = 0%).

These findings suggest that the oxygenation benefit of HFNOT may be more evident in shorter-duration applications and in patients undergoing non-cardiothoracic procedures.

## 4. Discussion

In this systematic review and meta-analysis, we evaluated the efficacy of HFNOT in reducing post-extubation hypoxaemia and postoperative pulmonary complications compared to COT across various surgical populations. Seventeen trials were included, comprising a range of procedures including cardiac, thoracic, abdominal, orthopaedic and bariatric surgeries. Our findings indicate that HFNOT significantly improves oxygenation, reduces the incidence of postoperative pulmonary complications, and is associated with lower rates of reintubation, hospital length of stay and postoperative mortality. However, no statistically significant differences were found in ICU length of stay.

These results align with existing evidence from critical care settings, where HFNOT has been shown to enhance oxygenation and reduce respiratory distress in patients with acute hypoxaemic respiratory failure. Our findings expand the scope of HFNOT’s benefits into the perioperative context, suggesting its potential as a prophylactic intervention in post-extubation care, particularly in patients undergoing non-cardiothoracic surgeries.

Subgroup analyses provided further insights. Interestingly, the three studies in which HFNOT was applied for more than 24 h involved patients undergoing lung resection, major upper abdominal, and cardiothoracic surgeries—all of which are associated with a higher baseline risk for postoperative pulmonary complications. These procedures, including the effects of cardiopulmonary bypass, inherently compromise respiratory mechanics through direct lung injury as well as impaired diaphragmatic function and chest wall compliance. As such, the need for prolonged oxygen therapy in these trials may reflect greater illness severity or slower postoperative recovery. Furthermore, variability in postoperative care protocols and timing of outcome measurements could have attenuated the observed benefit in this subgroup. As such, the physiological benefits of HFNOT may be most impactful in the early postoperative period, and prolonged application may not yield additional improvement in oxygenation metrics or reduction in postoperative pulmonary complications. Similarly, when stratified by surgical type, patients undergoing non-cardiothoracic surgeries exhibited a greater improvement in PF ratio than those who had cardiothoracic procedures. The diminished benefit in the latter group may be due to the confounding influence of factors such as cardiopulmonary bypass and fluid shifts that independently affect postoperative lung function.

Previous meta-analyses assessing the efficacy of HFNOT in various clinical scenarios have been published, including those with obesity in the perioperative period, post-extubation adult surgical patients, post-cardiothoracic surgeries and patients who were at high risk for PPC [37,38,39]. Of these studies, the majority of them analysed patients post-cardiothoracic surgeries as they are more prone to pulmonary complications and respiratory failure. The studies of Wang et al., Zhu et al. and Wu et al. showed a significant reduction in the escalation of respiratory support but only the study of Wang et al. reported a reduction in reintubation rate [40,41,42]. No meta-analyses showed an improvement in ICU length of stay, as similarly reported in our study.

HFNOT’s physiological advantages include the delivery of heated and humidified high-flow oxygen, generation of low-level positive airway pressure, reduction in dead space, and improved mucociliary clearance and likely contribute to its effectiveness. These mechanisms support better ventilation–perfusion matching and reduced work of breathing, which may explain the observed clinical benefits.

The strengths of this meta-analysis include a comprehensive and systematic literature search, inclusion of only randomized controlled trials, and detailed subgroup analyses. It also has the highest number of RCTs compared to previous systematic reviews and meta-analyses of this topic. These approaches enhance the reliability and generalizability of our findings across a broad surgical population.

However, several limitations must be acknowledged. Notable heterogeneity was observed in several outcomes, particularly in PF ratio and hospital length of stay, which may reflect differences in HFNOT protocols, surgical types, and clinical settings. Inconsistent definitions of postoperative pulmonary complications and variable thresholds for escalation of care could have introduced bias. Furthermore, all trials were unblinded to the patient and primary doctor and some did not clearly report on adherence to intervention protocols or the criteria for clinical escalation. Certain data needed formula conversion from median and interquartile range to mean and standard deviation, which may affect the normality of results. We also excluded data that was presented in graphical form as we did not obtain a reply from corresponding authors and the reverse-engineered numerical values might not be accurate.

These findings have important clinical implications. HFNOT may be considered a viable prophylactic respiratory support strategy following extubation in selected surgical patients, especially where ICU resources are limited or where patient comfort is prioritized. Its greatest impact appears to be in the early postoperative period and in patients not undergoing cardiothoracic surgery. Future research should focus on defining the optimal timing, duration, and patient selection criteria for HFNOT use in surgical populations. Longitudinal studies examining long-term outcomes and cost-effectiveness are also warranted.

## 5. Conclusions

This systematic review and meta-analysis supports the role of HFNOT in improving oxygenation and reducing respiratory complications after extubation in surgical patients. The most notable benefits were observed in non-cardiothoracic populations and during short-duration applications, underscoring the importance of tailored perioperative respiratory strategies.

## Figures and Tables

**Table 1:** Baseline characteristics of included trials.

Study ID, Location	Inclusion Criteria	Clinical Setting	Target SpO_2_	Number of Patients Total (H/C)
Allam [19], Egypt	Age 18–60 BMI > 40 Preop ASA III Postop atelectasis Laparoscopic sleeve gastrectomy	Bariatric surgery	N/A	110 (55/55)
Brainard [20], United States	Age > 18 Postop thoracic surgery	Thoracic surgery	90	44 (18/26)
Burra [21], India	Age 18–65 BMI < 30 Preop ASA < III Elective cardiac surgery	Cardiac surgery	N/A	60 (30/30)
Corley [22], Australia	Age > 18 BMI > 30 Cardiac Surgery on cardiopulmonary bypass	Cardiac Surgery	95	155 (81/75)
Ferrando [23], Spain	Age > 18 BMI > 35 ASA II–III Laparoscopic bariatric surgery	Bariatric surgery	N/A	64 (32/32)
Frassanito [24], Italy	Age > 18 Female BMI < 35 Laparoscopic gynae surgery > 2 h	Gynaecological Surgery	94	83 (42/41)
Fulton [25], Australia	Age > 18 BMI > 30 Laparoscopic bariatric surgery	Bariatric surgery	95	50 (25/25)
Futier [26], France	BMI < 35 Surgery > 2 H Intermediate–High-risk PPC	Abdominal Surgery	95	220 (108/112)
Li [27], China	Age > 65 ASA I–III	Orthopedic surgery	90	60 (30/30)
Parke [28], New Zealand	Age > 18 Surgery with full median sternotomy	Cardiac surgery	93	340 (169/171)
Pennisi [29], Italy	BMI < 35 Elective thoracotomic pulmonary lobar resection	Thoracic surgery	92	95 (47/48)
Sahin [30], Turkey	Age > 18 BMI > 30	Cardiac surgery	93	100 (50/50)
Soliman [31], Egypt	Age 50–70 BMI < 35 ASA I–III Major elective upper abdomen procedures	Upper abdominal surgery	94	80 (40/40)
Sun [32], China	BMI > 18 and < 30 Robotic-assisted laparoscopic rectal cancer surgery	Colorectal surgery	95	78 (39/39)
Theologou [33], France	Age > 18 Spontaneous Breathing Trial PF ratio < 200 Elective or urgent cardiac surgery	Cardiac surgery	92	99 (66/33)
Vourc’h [34], France	SpO2 < 96% after extubation with Venturi mask FiO_2_ 0.5	Cardiac surgery	96	82 (41/41)
Yu [35], China	Age 18–80 Intermediate–High-risk PPCs Thoracoscopic lobectomy for lung tumour	Thoracic surgery	95	110 (56/54)

H = HFNOT group: N/A= Not Available; C = COT group; Chronic Obstructive Pulmonary Disorder (COPD); ASA Physical Status Classification System

**Table 2:** Details of study intervention.

Study ID, Location	Duration of Intervention	Intervention Details (Flow and FiO_2_)	Control Details	Therapy Algorithm	Outcomes
Allam [19], Egypt	24 h	30 L/min FiO_2_ 60%	VM FiO_2_ 60%	/	①③
Brainard [20], United States	48 h	40 L/min	NP or FM		②③④
Burra [21], India	4 h	60 L/min	NP 4 L/min		③⑤
Corley [22], Australia	5 days	35–50 L/min	NP 2–4 L/min or FM 6 L/min		①③⑤
Ferrando [23], Spain	3 h	60 L/min FiO_2_ 50%	VM 15 L/min FiO_2_ 50%	/	①②③⑤
Frassanito [24], Italy	2 h	60 L/min FiO_2_ 30%	VM 35%	/	①②⑤
Fulton [25], Australia	6 h	50 L/min FiO_2_ 50%	FM 6 L/min	/	①③④
Futier [26], France	24 h	50–60 L/min	NP or FM		②③④⑤
Li [27], China	1 h	40 L/min FiO_2_ 60%	FM 2 L/min O_2_ + 2 L/min air		②
Parke [28], New Zealand	72 h	45 L/min	NP or FM 2–4 L/min		③④
Pennisi [29], Italy	48 h	50 L/min FiO_2_ 40 ± 5%	VM 8 L/min ± 1		①②③④⑤
Sahin [30], Turkey	48 h	25–40 L/min FiO_2_ 50%	FM 2–4 L/min		②③④⑤
Soliman [31], Egypt	48 h	35–60 L/min	FM 6–10 L/min		①②③④
Sun [32], China		30 L/min FiO_2_ 50%	NP 4 L/min		②③④
Theologou [33], France	48 h	60 L/min FiO_2_ 60% 40 L/min FiO_2_ 60%	VM 15 L/min FiO_2_ 60%	/	②③④⑤
Vourc’h [34], France	48 h	45 L/min FiO_2_ 100%	HFFM 15 L/min	/	①③⑤
Yu [35], China	72 h	35–60 L/min FiO_2_ 45–100%	NP or FM		②③④⑤

① = post-extubation hypoxaemia (PF ratio), ② = Incidence of PPC, ③ = ICU Length of Stay, ④ = Hospital Length of Stay, ⑤ = Mortality.

**Table 3:** Escalation of Respiratory Support Among Patients Receiving HFNOT and COT.

Study	Group	*N*	Escalation to HFNOT	Escalation to NIV	Reintubation	Total Escalations (%)	
Corley [22]	HFNOT	81	0	3	0	3	3.7
	COT	75	3	1	1	5	6.7
Fulton [25]	HFNOT	25	0	0	0	0	0
	COT	25	1	0	0	1	4.0
Futier [26]	HFNOT	108	N/A	N/A	20	20	18.5
	COT	112	N/A	N/A	14	14	12.5
Parke [28]	HFNOT	169	7	9	2	18	10.7
	COT	171	12	5	0	17	9.9
Pennisi [29]	HFNOT	47	0	1	1	2	4.3
	COT	48	0	3	1	4	8.3
Sahin [30]	HFNOT	50	0	6	0	6	12.0
	COT	50	0	11	4	15	30.0
Soliman [31]	HFNOT	40	0	1	0	1	2.5
	COT	40	0	3	2	5	12.5
Vourc’h [34]	HFNOT	41	0	13	3	16	39.0
	COT	41	0	24	1	25	61.0
Yu [35]	HFNOT	56	0	2	0	2	3.6
	COT	54	0	9	5	14	25.9

N/A = Not Available.

**Table 4:** Subgroup Analysis of PF Ratio by Duration of Intervention and Surgical Type.

Outcome	No of Studies	HFNOT (*n*)	COT (*n*)	Mean Difference (95% CI)	*p*-Value	I^2^
Duration of intervention						
Less than 24 h	4	154	154	45.45 [27.92, 56.97]	<0.00001	86
More than 24 h	3	128	129	10.51 [0.26, 20.75]	0.04	26
Type of surgery						
Cardiothoracic	2	88	89	6.37 [−5.00, 17.75]	0.27	0
Non-cardiothoracic	5	194	193	38.50 [26.13, 50.88]	<0.00001	83

## Data Availability

The data is contained within the article.

## Supplementary Materials

The following supporting information can be downloaded at https://www.mdpi.com/article/10.3390/diagnostics15192449/s1, Supplementary File S1: PRISMA checklist. Reference [43] are citied in the Supplementary Materials.

## Abbreviations

The following abbreviations are used in this manuscript:

BiPAP	Bilevel positive airway pressure
BMI	Body mass index
χ^2^	Chi-squared
CI	Confidence interval
COPD	Chronic obstructive pulmonary disorder
COT	Conventional oxygen therapy
CPAP	Continuous positive airway pressure
EPCO	European perioperative clinical outcome
FiO_2_	Fraction of inspired oxygen
HFNOT	High-flow nasal oxygen therapy
I^2^	I-squared
IQRs	Interquartile ranges
ICU	Intensive care unit
MDs	Mean differences
NIV	Non-invasive ventilation
ORs	Odds ratios
RCTs	Randomized controlled trials
PEEP	Positive end-expiratory pressure
PF	PaO_2_/FiO_2_ ratio
PPCs	Postoperative pulmonary complications
PRISMA	Preferred Reporting Items for Systematic Review and Meta-analyses
SpO_2_	Oxygen saturation
